# Dietary (Poly)phenols and the Gut–Brain Axis in Ageing

**DOI:** 10.3390/nu16101500

**Published:** 2024-05-16

**Authors:** Léonie Láng, Simon McArthur, Alpar S. Lazar, Line Pourtau, David Gaudout, Matthew G. Pontifex, Michael Müller, David Vauzour

**Affiliations:** 1 Norwich Medical School, Biomedical Research Centre, University of East Anglia, Norwich NR4 7TJ, UK; leonie.lang@uea.ac.uk (L.L.); michael.muller@uea.ac.uk (M.M.); 2 Faculty of Medicine & Dentistry, Queen Mary, University of London, Blizard Institute, London E1 2AT, UK; s.mcarthur@qmul.ac.uk; 3 Faculty of Medicine and Health Sciences, The Queen’s Building, University of East Anglia, Norwich NR4 7TJ, UK; a.lazar@uea.ac.uk (A.S.L.); m.pontifex@uea.ac.uk (M.G.P.); 4 Activ’Inside, 33750 Beychac et Caillau, France; l.pourtau@activinside.com (L.P.); d.gaudout@activinside.com (D.G.)

**Keywords:** flavonoids, phenolic compounds, microbiome, neuroprotection, brain health, Alzheimer’s disease, dysbiosis

## Abstract

As the population ages, the incidence of age-related neurodegenerative diseases is rapidly increasing, and novel approaches to mitigate this soaring prevalence are sorely needed. Recent studies have highlighted the importance of gut microbial homeostasis and its impact on brain functions, commonly referred to as the gut–brain axis, in maintaining overall health and wellbeing. Nonetheless, the mechanisms by which this system acts remains poorly defined. In this review, we will explore how (poly)phenols, a class of natural compounds found in many plant-based foods and beverages, can modulate the gut–brain axis, and thereby promote neural health. While evidence indicates a beneficial role of (poly)phenol consumption as part of a balanced diet, human studies are scarce and mechanistic insight is still lacking. In this regard, we make the case that dietary (poly)phenols should be further explored to establish their therapeutic efficacy on brain health through modulation of the gut–brain axis, with much greater emphasis on carefully designed human interventions.

## 1. Introduction

An increase in the average age of the population is closely correlated with a similar increase in age-related diseases, including those affecting the brain [1]. A staggering 152.8 million cases of dementia are expected worldwide by 2050, having detrimental effects on individuals, their families, and the global economy [2,3]. Consequently, it is vital to understand how to support brain health and to develop strategies to delay cognitive decline during ageing, improving the quality of life of individuals, and addressing a major public health challenge.

The dynamic, bi-directional communication between the gut and the brain, also known as the “gut–brain axis” (GBA) has been the centre of extensive research in recent years. The GBA refers to the communication between the brain and the gut microbiota via multiple different pathways, as reported by us and others [4,5,6,7,8,9]. In addition, the functioning of the nervous system is affected by both the immune and the metabolic state of the body, with the gastrointestinal tract (GI) playing a major modulatory role [10,11].

The human GI tract is a rich and diverse ecosystem enabling several important physiological roles such as the maintenance of the host immune system and the integrity of the gut barrier through the production of various endogenous metabolites. For example, the gut microbiota may produce metabolites directly from the food ingested (e.g., short chain fatty acids; SCFAs), modify host-related metabolites (e.g., secondary bile acids), or produce de novo metabolites (e.g., polysaccharide) [12,13]. Indeed, the metabolism of different food components provides a wide range of by-products that can directly positively or negatively affect host health [14]. Moreover, the metabolic state of the organism also impacts the production of gut-derived metabolites, mainly through varying substrate availability, intestinal transit time [15] or redox balance [16], ultimately contributing to the overall homeostasis of the organism [17,18]. 

Dietary plant-derived (poly)phenols are an integral part of the human diet and can modulate the composition of the gut microbial community [17,19]. This interaction between gut microbes and metabolites of food-derived compounds appears to positively impact on human chronic diseases [20,21] and be a highly relevant modifier of brain physiopathology [22,23]. Consequently, research in the field of the GBA is now investigating how the gut microbiota can be a target for nutritional and therapeutic strategies to improve brain health during ageing. This review will explore the impact of (poly)phenols on the gut–brain axis and their potential role in promoting overall brain health.

## 2. The Microbiota–Gut–Brain Axis and Ageing

The mounting interest in the gut microbiota and its role as a contributing factor in health and disease has highlighted its potential impact on various pathologies, including neurodegenerative diseases. The gut microbiota is a concentrated population of 10^10^ to 10^11^ microbes per wet-weight gram of faeces [24], although it may be underestimated due to the presence of other microbes in the intestinal mucosa and other faecal fluids [25]. It is comprised of bacteria, archaea, fungi, and viruses, interacting with their host, affecting many important functions such as nutrient metabolism, xenobiotic and drug metabolism, maintenance of structural integrity of the gut barrier, immunomodulation, and protection against pathogens [26]. Its composition is highly variable, although a number of key bacterial species are typically present in most individuals. Several factors influence this variability, with the type of birth (vaginal vs. caesarean section) to diet being the most impactful [27,28,29]. An imbalance in microbial community structure, sometimes termed as gut dysbiosis (almost wrongly, as the healthy/eubiotic microbiome is not yet defined) or the lack of colonisation resistance (resilience to the invasion of pathogens) [30] can encourage the growth of pathobionts which will compete for nutrients, modify metabolite production or enhance immune responses [31]. Pathogenic bacteria can cause inflammation by altering the gut environment and exploit compounds derived from the commensal microbes for their own growth. Increased intestinal permeability, enabling diffusion of pathogenic bacteria into the systemic circulation and ultimately the brain, may lead to synaptic dysfunction and neuronal loss, eventually affecting cognitive and memory function [32]. Taken together, these processes may contribute to pathological brain ageing and eventually Alzheimer’s disease (AD) [33]. 

### 2.1. The Gut Microbiome and Ageing

**Figure 1:**
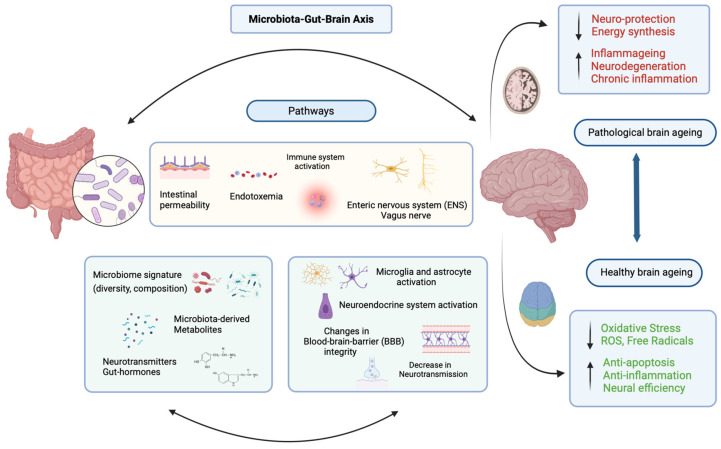
The microbiome gut–brain axis and its importance in modulating brain health during ageing. Overview of pathways through which the gut microbiota interacts with the brain, the factors influencing microbiome composition, and the resulting effects on brain health. The gut microbiome can influence intestinal permeability and modify gut barrier integrity. Increased gut permeability can lead to the presence of endotoxin in the blood stream (endotoxemia), activate the immune system and influence systemic immune responses. Direct communication channels between the gut and the brain are occurring for example via the Enteric Nervous System (ENS) and the *vagus nerve* among others. The composition of the gut microbiota can influence neurotransmitters and gut hormones levels, as well as the levels of gut-derived neuroprotective metabolites, and impact signalling pathways involved in cognitive functions and neuroactivity. Dysregulation of the microbiota–gut–brain axis can lead to neuroprotection deficits, reduction in energy synthesis and neurodegeneration (microglia and astrocyte activation), all characteristics of a pathological brain. On the other hand, proper functioning of the gut–brain axis can counteract oxidative stress, reduce reactive oxygen species (ROS) and free radicals, promote anti-apoptotic mechanisms, enhance anti-inflammatory responses, and improve neural efficiency, thus supporting a healthy brain during ageing (created with BioRender.com (https://biorender.com/, accessed on 21 April 2024).

The impact of the microbial composition and associated changes suggests that microbes can accelerate the ageing process [34]. Biological vulnerability and decreased physiological reserves associated with neurobiological changes such those observed in age-related frailty [35], collocate with a reduction in gut microbiome diversity in the elderly [36,37]. Research has highlighted a distinct change in the gut microbiome profile of centenarians, characterised by a rearrangement of the Firmicutes population and an enrichment in facultative anaerobes, including potential pathobionts [38]. This change in microbial composition may have a negative impact on the host, particularly due to the under-representation of species known to produce SCFAs such as butyrate, which are essential for stimulating epithelial cell growth and promoting gastrointestinal health [39]. Studies have also explored the association between specific age-related traits, such as frailty, and the functional profile of the gut microbiome in rodents [40]. Findings reveal that ageing may be associated with changes in taxonomic and functional patterns, including the under-maintenance of specific host nutrition-related functions [41,42]. More recently, increasing microbiome compositional uniqueness was shown to both reflect healthy ageing and predict lifespan in older adults [43]. This increase in uniqueness is accompanied by a decline in the core genus Bacteroides, marking it as a characteristic of healthy ageing. Furthermore, studies of faecal microbiome repository data across five continents and with participant ages ranging from 18 to 107 years old showed that gut microbiome alterations associated with both typical and pathological ageing are likely characterised by a loss of the stable components of the microbiome and concomitant increase in disease-associated taxa [44] (Figure 1).

### 2.2. Gut Microbiota, Inflammation, and the Blood-Brain Barrier

One of the body’s natural defence systems to fight external stressors is inflammation. Although beneficial in the short term, persistent low-grade inflammation may fuel the development of neurodegenerative diseases and is accompanied by a parallel systemic immune dysfunction [45]. Low-grade chronic inflammation, also termed ‘inflammageing’, is promoted by both endogenous and exogenous triggers, with the gut microbiota now recognised as a key player [46]. Controlling, slowing or reversing low-grade inflammation is likely to be an important approach to prevent or minimise the severity of age-related functional decline, including in the brain, and the onset of conditions which adversely affect health and well-being. In addition to inflammation, central to brain health is the control of influx and efflux of biological substances essential for brain metabolism and neuronal function, processes ensured by the blood-brain barrier (BBB) [45]. The main role of the BBB is to protect the brain from toxic substances in the blood and to act as a filter for nutrients [47], but when its integrity is damaged, it is thought to permit and contribute to neuropathological processes. Pathogenic bacteria can invade the brain through a variety of complex putative mechanisms [48], a feature that may be affected by gut microbial changes. Homeostasis of the gut microbiome can ensure better gut barrier integrity, thereby limiting bacteraemia, systemic inflammation and ultimately potential brain invasion by pathogens. The absence of a commensal microbiota certainly influences BBB development, disrupting expression of the tight junction proteins crucial for optimal BBB efficacy [49] (Figure 1). Interestingly, BBB disruption was partly restored following either colonisation of germ-free mice with a conventional murine microbiota or upon feeding with a sodium butyrate solution. Other gut-derived metabolites such as trimethylamine *N*-oxide (TMAO), *p*-cresol conjugates and propionate have also been reported to exert protective effect upon the BBB [50,51,52] which further complicate our understanding of the bacterial-host interactions in healthy and pathological ageing.

### 2.3. The Gut–Brain Axis and Neurological Disorders

The autonomic nervous system plays a significant role in gut–brain communication, with both the parasympathetic *vagus nerve* and multiple sympathetic efferent fibres connecting and regulating endocrine, immune, humoral, and gastrointestinal functions. The *vagus nerve* represents a particularly important link between nutrition and neurological and inflammatory diseases, with vagal activity having been shown to modulate the enteric nervous system (ENS) and the gastrointestinal tract, with the gut microbiota playing a mediatory role [53,54]. Notably, alterations in the ENS occur prior to CNS manifestations of pathology, suggesting a potential ‘‘anticipatory” pathological communication with the brain [55]. Moreover, the ENS can be potently influenced by gut microbes, such that disorders in the gut microbes can prime disorders in the ENS. Given that disturbances in the ENS correlate with disorders in the CNS, it is unsurprising to observe GI issues in diseases impacting the nervous system and neurological disorders [56]. Indeed, in some neurological diseases, particularly Parkinson’s disease, peristalsis is compromised resulting in constipation and GI symptoms occurring in 60 to 80% of patients [57,58,59]. 

### 2.4. Diet, Gut Microbiota, and Gut–Brain Communication

Diet plays an important role in gut–microbiota and gut–brain communication, but these interactions are complex and multi-faceted [60]. Indeed, diet composition and the gut microbiome regulate production of a broad range of metabolites [61], but the potential influence of these compounds on cognitive health are only beginning to be uncovered. For example, although short chain fatty acids, secondary bile acids, TMAO, tryptophan derivatives, cresols and other metabolites have been reported to affect cognitive function and neurodegenerative disorders [22,61], this represents only a small fraction of the known microbe-derived molecules in the circulation. Thus, the microbiota and diet are critical modulators of gut–brain communication (Figure 1). 

## 3. (Poly)phenols, Gut–Microbiota–Brain Axis and Ageing

Plant-derived (poly)phenols, a group of naturally occurring phytochemicals present in high amounts in fruits, vegetables and cereals, in addition to their impact on brain functions [62], have been reported to impact the intestinal microbiota by supporting the proliferation of beneficial microbes or by suppressing the proliferation of pathogens [63]. Indeed, a “prebiotic-like” effect of phenolic compounds and (poly)phenol-derived gut microbial metabolites has been reported to increase protective strains of bacteria, to decrease pathogenic inflammatory microorganisms, and to influence the Firmicutes/Bacteroidetes ratio, that is altered with ageing [17,64]. However, the different biological activities of various (poly)phenols will depend both on their bioavailability, itself influenced by factors such as age, sex and body composition, and on their secondary metabolites produced by the gut microbes [60,61], potentially explaining inter-individual variability in response to (poly)phenol intake [65]. Evidence that interactions exist between (poly)phenols and brain health, mediated via effects on the gut microbiome and inflammatory pathways, is compelling and widely reported [66], but whether products of polyphenol-mediated gut microbial metabolism are associated with brain activity is far less well-established [67].

With the aim of capturing the current evidence of the role of (poly)phenols on the gut–brain axis and their impact on brain health, we conducted a non-systematic comprehensive literature search, using keywords related to brain health and cognitive status, polyphenolic compounds, gut microbiome composition and metabolites related to brain health. This search resulted in 462 hits up to 9 November 2023. Following a first screen based on title and abstract readings, the full text screening resulted in 56 studies (53 preclinical and 3 clinical) that were included in this review and are detailed below. The results retrieved are presented in the Table 1 for preclinical studies and in the Table 2 for clinical studies.

### 3.1. Preclinical Evidence

#### 3.1.1. (Poly)phenols, Gut Microbiome Modulation and Related Brain Health

The significant role of (poly)phenols in modulating the gut microbiome and their potential benefits for brain health and cognitive functions is evident through various studies on flavanols, flavonols, flavones, phenolic compounds, polyphenol-rich extracts and other botanicals (Table 1). These studies have demonstrated that (poly)phenols such as epigallocatechin gallate (EGCG) and epicatechin (EC) can increase both bacterial and species richness, decrease harmful bacteria such as Proteobacteria, and increase the abundance of beneficial bacteria such as *Akkermansia* and *Bifidobacterium* [68]. The modulation of the gut microbiome is associated with decreased activation of the microglia and inflammatory pathways in the brain, suggesting a protective effect against neuroinflammation and cognitive decline. Similarly, flavonols (e.g., quercetin) have been reported to increase the abundance of beneficial gut bacteria, such as *Barnesiella* and *Lactobacillus*, while decreasing the detrimental taxa *Alistipes* and *Rikenella* (Bacteroidetes phylum) [69]. These changes in the gut microbiome are linked with improved spatial memory, decreased amyloid-beta (Aβ) accumulation, and tau phosphorylation in mice, indicating potential benefits for AD prevention. Phenolic compounds such as curcumin and sesamol have also been associated with alterations in gut microbiota composition, leading to improved spatial learning and memory, decreased Aβ pathology in the hippocampus, and reduced neuroinflammation [70,71,72] suggesting neuroprotective effects of these compounds. Moreover, other (poly)phenols including fisetin [73], icariin [74] and various flavones such as baicalein [75,76] and apigenin [77] have also been reported to improve behavioural impairments, enhance motor learning and coordination, and decrease neuroinflammation through modulation of the gut microbiota composition and microbial metabolites. (Poly)phenol-rich extracts and botanicals, such as blackberry and blueberry anthocyanin-rich extracts [78,79], have been shown to restore gut dysbiosis, increase the production of SCFAs, improve cognitive performance and improve antioxidant activities in the brain. Overall, (poly)phenols improve gut microbiome composition by increasing microbial abundance and diversity and by restoring a gut microbial balance between the Firmicutes/Bacteroidetes ratio [74,80,81,82].

#### 3.1.2. Insight into Associated Molecular Mechanisms

**Figure 2:**
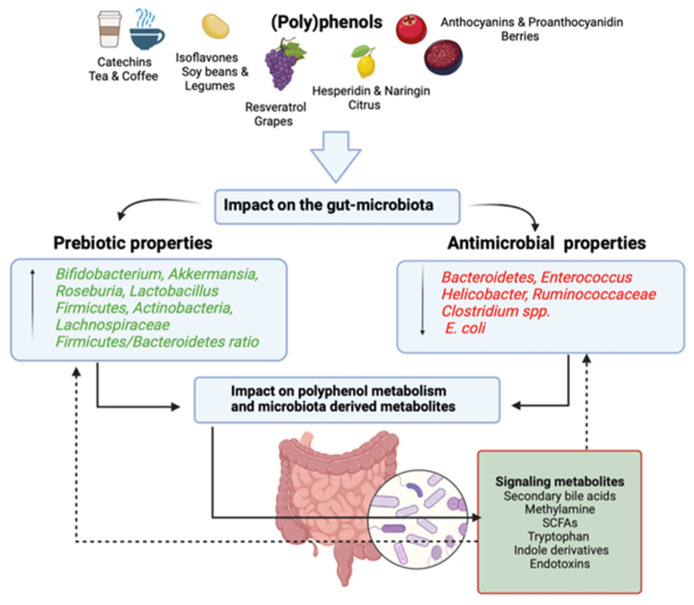
Impact of (poly)phenols on gut microbiota. The activities of the diverse (poly)phenol sources and their impacts on the gut microbiota of which prebiotic and antimicrobial properties, polyphenol metabolism and microbial-derived metabolites influence the gut ecosystem (created with BioRender.com).

The positive impact of (poly)phenols on the gut microbiome are in turn linked with improved cardiometabolic functions, reduced immune activation and secretion of anti-inflammatory molecules, and a tangible positive effect on the intestinal barrier. In particular, (poly)phenols modulate the expression/regulation of key genes/pathways related to antioxidant activities, maintenance of the BBB integrity, and overall brain functions, including the regulation of astrocytes and microglia activities. These beneficial changes are most often correlated with increased relative abundance of Bacteroides, *Akkermansia*, *Lactobacillus*, *Lachnospiraceae*, *Ruminococcus*, *Muribaculaceae*, and *Bifidobacterium* (Figure 2).

These observations include an increase in neurotrophic factors such as BDNF, a down-regulation of inflammatory pathways and a reduction in pro-inflammatory cytokines (IL-1β, IL-6, and TNF-α) and endotoxins (LPS). The effects of (poly)phenol on the gut microbiome and neurological health, including major disease-related aspects such as the reduction in pathological protein aggregation, the restoration of hippocampal neuronal plasticity or increased expression, which are key regulators of barrier integrity [77,83,84,85,86]. The interaction between (poly)phenols and LPS occurs in multiple ways, either by inhibiting the expression of pro-inflammatory cytokines and LPS-induced mediators [87], suppressing TLR4/NF-κB pathways [87,88], attenuating LPS-induced oxidative stress [89] or even by binding LPS directly, therefore preventing its interaction with cellular receptors [90]. However, the response to LPS is dose-dependent, and health effects vary according to its exposure. High levels of LPS can cause endotoxemia, leading to septic shock and severe human health consequences [91,92]. Consequently, the beneficial effects of (poly)phenols from this angle should be taken with reserve when assessing their effects, particularly under acute conditions.

In addition, reduction in oxidative stress mediated by improved antioxidant defence and increased enzymatic activity (e.g., superoxide dismutase (SOD) or catalase (CAT)) is also commonly reported in pre-clinical studies. Neuronal tissues have a high metabolic rate, and therefore produce elevated amounts of reactive oxygen species (ROS) when compared to other organs [93]. For example, during the mitochondrial oxidative phosphorylation, anion superoxide is generated as a by-product of ATP, and transformed into oxygen. Both commensal and pathogenic gut bacteria have the capacity to modulate mitochondrial activity, and therefore to alter ROS production [94]. In addition, metabolites such as SCFAs help to regulate redox homeostasis by influencing mitochondrial activity [95] via the induction of signalling pathways such as the nuclear erythroid 2-related factor 2 (Nrf2) which governs antioxidant synthesis and enzymes involved in the elimination of ROS [96].

When the gut homeostasis is compromised, anti-inflammatory molecules such as SCFAs are decreased and an imbalance occurs with levels of pro-inflammatory molecules (e.g., LPS or biofilm amyloid fibres) [97,98] resulting in greater permeability of the intestinal wall, increased mitochondrial ROS production, neuronal peroxisome proliferation, as well as neurotoxin aggregation and, potentially, neurodegeneration. The decline in free radicals in the brain, through the reduction of oxidation by the modulation of the gut microbiome with the consumption of (poly)phenolic compounds, is reflected by the decrease in the level of malondialdehyde (MDA) and ROS, which is concordant with the changes in SCFAs production or the increase in SCFA-producing bacteria in several studies [74,80,82,85,99,100,101,102,103,104,105,106] (Table 1). This interplay between gut microbiota and brain oxidative stress highlights the potential of the gut microbiome to modulate the brain redox status. Furthermore, there is also a consensus regarding (poly)phenol consumption, the gut microbiome ecosystem and brain integrity, via the maintenance of BBB, improvement of synaptic activity, and the overall reduction of inflammation in the brain [68,77,80,107,108]. In some cases, there is a parallel between the observed increase in SCFA levels, or the increase in SCFA-producing bacteria and a positive regulation of astrocyte and microglial activity in the brain, hence collectively supporting neuronal activity [68,107,108]. 

The release of specific hormones (corticotropin, adrenocorticotropin, corticosterone or glucocorticoids and other hormones) highlights the neuroendocrine activity of (poly)phenolic compounds in addition to their neuroprotective effects. Indeed, it seems that the neuroendocrine effects of (poly)phenols could be responsible for at least part of their neuroprotective effects, as it was reported with regard to the free radical scavenging effects of oestrogen (as well as genomic effects) and the consequent neuroprotection. This is notably illustrated by an a posteriori increase in neuroinflammation, which was observed following menopause and the associated fall in oestrogen levels [109,110]. In models of (neuro)inflammatory diseases, e.g., direct administration of amyloid beta Aβ1–42 peptide [111], D-galactose [68,80,81,82,99,104,105] or by other neurotoxins [111], oxidative stress, inflammation and mitochondrial dysfunction occur, usually in parallel to an abnormal gut ecosystem. This is of major importance as it validates the bidirectionality of the gut–brain axis, with induced neuro-disorders causing deleterious changes at the level of the microbiome. A consensus between the related homeostatic dynamics of the gut microbiome with (poly)phenols and the decreased in inflammasome and overall brain inflammation (reduced cytokines levels and macrophage activities) along with overall oxidative status is also evident. This is a determining factor as it validates the mediating effects of the gut microbiome structure towards brain health and antioxidative properties, while confirming the prebiotic effects of (poly)phenolic compounds (Table 1). 

**Table 1:** The effects of (poly)phenols on gut microbiome structure, microbial metabolites, and related brain functions in models of neurological diseases and ageing: Results from preclinical studies.

Polyphenol Type	Study Model, Sample Size, Dose, and Duration	Changes in Microbiome Structure	Changes in Microbial Metabolites	Functions	Refs.
Isolated compounds					
Flavanols					
Epigallocatechin gallate (EGCG)	Female Sprague-Dawley (SD) rats (*n* = 60), 75 mg/kg, 150 mg/kg, and 300 mg/kg, 12 weeks	↑ bacterial and species richness, ↓ *Proteobacteria*. ↑ abundance of *Akkermansia* and *Bifidobacterium*. ↓ *Enterococcus* and *Escherichia-Shigella*	NC	↓ activation of microglia (↓ Iba-1 positive cells in hippocampus), ↓ TLR4/NF-κB signalling pathway (TLR4 and IRAK proteins) and ↓ IL-1β, IL-6 and TNF-α. ↓ LPS levels	[68]
Epigallocatechin gallate (EGCG)	Male C57BL/6 mice, GTP 0.1% (*w*/*w*), 4 weeks	↑ *Bacteroidetes* and *Actinobacteria*. ↑ abundance of *Lactobacillus*. ↑ intestinal flora species	↑ metabolic levels of daidzein and *O*-DMA, O-acetyl -L-carnitine, trans-caffeic acid and daidzein	↑ regulation of astrocytes and oligodendrocytes, adjusted expression of core clock genes (Csnk1d, Clock, Per3, Cry2, and BhIhe41), ↑ lipid and amino acid metabolism	[107]
Eigallocatechin-3-gallate (EGCG)	Drosophila melanogaster male, *n* = 60, 0.1 mM or 0.5 mM EGCG, 3 days and 20 days	Normalized the microbial diversity, ↓ *Proteobacteria*, *Acetobacter* and *Lactobacillus*	NC	Improved locomotive functions, rescued ↓ life span	[112]
Epicatechin (EC)	C57BL/6J mice (*n* = 50), 2 or 20 mg/kg body weight, 24 weeks	↑ *Firmicutes*, *Acidobacteria*, *Bacteroidetes* and *Nitrospirae* and ↓ *Actinobacteria*. ↑ alpha diversity (higher dose group)	Changed metabolomic profile (↑ organic acids, nucleotides, and fatty acid isobutyrate) ↓ Alanine, valerate, cytosine and citrate	No effects on recognition, spatial memory and learning, mitigated anxiety-related behaviour. ↑ BDNF mRNA levels (32% higher EC group), ↓ glucocorticoids in the brain. Correlation between metabolome, microbiome, and behaviour test (OF)	[113]
Flavonols					
Quercetin-3-O-glucuronide (Q3G)	Male C57BL/6J mice, *n* = 30, 50 mg per kg/day, 4 weeks	↑ *Barnesiella* and *Lactobacillus*; ↓ *Alistipes* and *Rikenella*	↑ Short-chain fatty acids (SCFAs)	Alleviated spatial memory impairment, ↓ Aβ accumulation, ↓ tau phosphorylation	[114]
Quercetin	Aged male ICR mice, *n* = 32, 0.08% quercetin, 21 days	↑ gut microbiota α-diversity, ↓ *Verrucomicrobia*, *Blautia* and *Anaerotruncus*, ↑ *Tenericutes*	NC	Partial reversed of dAGEs-induced cognitive impairment, ↓ protein expression related to A-Beta generation and tau phosphorylation (Cathesin B and *p*-Tauser396 & 404) ↓ reactive astrocytes	[100]
Quercetin	Sprague Dawley rats, *n* = 30 10 mL/kg or of 50 mg/kg, 12 weeks	↑ *Actinobacteria*, ↓ *Porphyromonadaceae*, *Oxalobacteraceae*, *Oxalobacter* and *Klebsiella*	NC	Prevented myelin and axonal damage, ↓ RO	[69]
Quercetin	Male C57BL/6 mice, *n* = 24 50 mg/kg, 7 days	↑ intestinal permeability, normalized microbiome abundance	↑ Short-chain fatty acids (SCFAs) acetate and propionate levels	↓ neuropsychiatric problems, ↑ expression of occludin and doublecortin in frontal cortex and hippocampus, ↑ level of this tight junction protein, ↓ anxiety-like behaviour	[83]
Fisetin	Male C57BL/6 mice, *n* = 24, 100 ng/kg/bw/d, 30 days	↑ *Lachnospiraceae*, ↓ *Escherichia- Shigella* and *Bacillus*	NC	Improved behavioural impairments, ↑ tyrosine hydroxylase	[73]
Icariin (*Herba Epimedii*)	Male C57BL/6J mice, *n* = 20 100 mg/kg, (15 days)	↑ epithelial inflammation, offer a distinct taxonomic profile related to young mice in the old mice, ↑ SCFA-producing bacteria. ↑ beneficial bacterial profile	NC	↑ motor learning and coordination in aged mice, ↑ SOD, ↓ H_2_O_2_ and ROS levels (Malonaldehyde (MDA) levels, ↑ Nrf2 activity ↓ expression of FOXO1 and ANXA3 in old mice	[74]
Flavones					
Baicalein, *Scutellariae baicalensis Georgi*	Male SAMP8 and SAMR1 mice, *n* = 42, 200 mg/kg/d, 6 weeks	↓ *Mucispirillum*, *Parabacteroides*, *Prevotella*, *Bacteroides*, and *Sutterella*, ↑ *Christensenellaceae*	NC	↓ grading score of senescence, ↑ cognitive functions, inhibited release of proinflammatory cytokines in the brain cortex. *Christensenellaceae* correlates positively with the recognition index	[75]
Baicalein, *Scutellariae baicalensis Georgi*	Male C57BL/6J mice, *n* = 135, 25,50 and 100 mg/kg, 7 days	Restored *Firmicutes* and *Bacteroidetes* ratio, ↑ *Halomonas_smyrnensis* ↓ *Parabacteroides_johnsonii* and *Bacteroides_uniformis*	↓ TMA and TMAO plasma levels	↑ recognition memory, spatial learning, and memory, ↑ brain functional connectivity, restored the hippocampal neuronal plasticity, ↓ pro-inflammatory cytokines	[76]
Isootientin (ISO)	Male PP/PS1 mice, *n* = 45, 25 mg/kg or 50 mg/kg, 2 months	Regulated abnormal gut microbiome, ↑ α-diversity in caecum. ↑ *Mollicutes*, *Prevotellaceae*, and *Prevotellaceae UCG 001* bacteria	NC	↓ β plaque deposition in cortex and hippocampus of AD Mice, TNF-α ↓, IL-6 ↓, IL-4 ↑, IL-10 ↑; iNOS ↓, COX-2 ↓, ROS ↓	[102]
Apigenin	Male Sprague Dawley rats, *n* = 36, 20 mg/kg, 10 days	Mitigated stress-induced dysbiosis of gut microbiota, regulated composition, and abundance of gut microbiota	NC	↑ intestinal barrier function (expression levels of occludin and ZO-1), reversed mast cell and microglial activation, inhibited the activation of mast cells, prevented microglial activation	[77]
Vitexin, millet-derived flavonoid	Male C57BL/6 mice, *n* = 24, Low dose 10 mg/kg, high dose 30 mg/kg + HF diet, 4 weeks	↑ α-diversity, reversed HF diet microbiome alterations, ↑ *Akkermansia*, ↓ *Lachnospiraceae*	NC	↓ expression level of inflammatory cytokines in brain and intestine (TNF-a and IL-1B), improve oxidative stress and blood lipids parameters. ↓ MDA levels, ↑ GHS	[101]
Phenolic compounds					
Curcumin	APP/PS1 double transgenic mice, *n* = 15, High group 200 mg/kg body weight, low group 50 mg/kg, 3 months	↓ *Bacteroidaceae*, *Prevotellaceae*, and *Lactobacillaceae* and ↑ *Rikenellaceae*	↑ demethylcurcumin (M1) and bisdemethoxycurcumin metabolites	↑ spatial learning and memory, ↓ Aβ pathology in the hippocampus	[70]
Curcumin	C57BL/6 mice, *n* = 30, 100 mg/g/day, 7 days	Restored levels of *Bacteroidetes* and *Deinococcus-Thermus*, ↑ *Muribaculaceae*	Changed metabolites related to glycerophospholipid metabolism, specific regulation of 1-butylimidazole and tryptophan by curcumin	Alleviated anxiety-like behaviours restored lipid metabolism, ↑ phosphatidylcholine in the prefrontal cortex	[71]
Sesamol, (*Sesamum indicum*)	Male C57BL/6 ApoE knockout mice (ApoE−/−), *n* = 60, 100 mg/kg/bw, 8 weeks	↑ gut bacteria producing short-chain fatty acids (SCFAs), ↑ *Bacillales*, *Fusobacterium* and *Lactococus*	↑ SCFA acetate	↑ synapse ultrastructure and inhibited Aβ accumulation, prevented gut barrier damages and systemic inflammation, ↑ cognition	[115]
Sesamol, (*Sesamum indicum*)	APPswe/PS1dE9 AD mice, *n* = 24, 100 mg/kg/bw, 8 weeks	Reshaped gut microbiota composition, ↑ *Rikenellaceae* and *Bifidobacterium* (slight change), ↓ *H. hepaticus*, *Clostridium*, and *Bacillaceae*	↑ SCFAs acetate, propionate, isobutyrate, butyrate, and valerate	Inhibited plaque deposition in cortex and hippocampal CA1, repressed expression of APP and β-secretase (Bace1), ↓ neuroinflammation (TNF-a and IL-B)	[72]
*p*-coumaric acid (PCA)	Male C57BL/6 J Low-dose (20 mg·kg^−1^), high-dose (40 mg·kg^−1^), 28 days	Corrected gut microbiota abnormalities	Regulated arachidonic acid, tyrosine metabolism, and unsaturated fatty acid biosynthesis, glycolysis/glycogenesis and glycerophospholipid (neuroinflammation and energy metabolism)	↓ Aβ1–42, *p*-Tau proteins in the brain, ↓ ROS and MDA, ameliorated neuroinflammation	[103]
Chlorogenic acid (CGA)	Male C57 BL/6J mice, *n* = 40, 30 mg/kg/day, 11 days	↑ *Lactobacillus*, *Firmicutes*, ↓ *Bacteroides microbiome*-related neurotransmitters	↓ kynurenic and quinolinic acid (KYN and Quin)	Alleviated TMT-induced epilepsy-like seizure and cognitive impairment, ↓ hippocampal neuronal degeneration and neuroinflammation. ↑ levels of SCFAs (propionic and isobutyric acid) in hippocampus. ↑ DL-kynurenine and acetylcholine chloride	[111]
Chicoric acid (CA) (*Echinacea purpurea*)	C57BL/6J mice, *n* = 60, 60 mg kg^−1^, 12 days	↓ microbial dysbiosis, ↓ *Bacteroidetes* and *Parabacteroide*, ↑ *Firmicutes*, *Lactobacillus* and *Ruminiclostridium*. ↑ *Lachnospiraceae*, *Lactobacillus*, *Riminiclostridium* and *Lachnoclostridium*	Restored normal SCFA production	Better motor performance, ↓ TNF-α and IL-1β in the serum, striatum and colon, ↓ neuroinflammation and gut integrity. ↑ expression of BDNF and GDNF, prevented neurotrophic suppression, Promoted colonic epithelial integrity	[116]
Corylin, *Psoraleae Fructus*	Female C57BL/6J mice, *n* = 72, low dose 10 mg/kg/day, medium 30 mg/kg/day, and high 90 mg/kg/day, 4 days	↓ *Bacteroides* and *Escherichia-Shigella*, ↑ *Enterorhabdus* and *Candidatic_Stoqueficus* and ↓ *Turicibacter*. Key bacterial type maintained by Corylin were *Muribacylaceae*, *Dubosiella* and *Lactobacillus*	NC	Maintained BBB structural and functional integrity, ↓ neuroinflammation and ↑ expression of TJ proteins. ↑ 5-HT, 5-HTP, and Trp levels. Dose dependent effects. Ameliorate colon damage and inflammatory response (↓ IL-6 and TNF-α), reversed bacterial composition and diversity	[108]
Stilbenes					
Resveratrol (Res)	C57BL/6 mice, *n* = 55, 30/mg/day, 8 weeks	↑ *Ruminoclostridium*, *Odoriabacter*, *Prevotellaceae*, *Rikenellaceae*, *Alistipes* and *Blautia*, ↓ Fimicutes/Bacteroidetes, ↓ *Lachnospiraceae*, *Ruminococcaceae*, *Lactobacillus*, *Lachnospiraceae* and *Akkermansia*.	NC	Alleviated mice phenotype from PD progression. ↑ motor functions, ↑ intestinal transit rate, alleviated dopaminergic neurodegeneration, ↓ relative abundance of inflammatory cytokines (TNF-α, IL-6 and IL-1β)	[117]
Polyphenols-rich extracts and Botanicals					
Blackberry anthocyanin-rich extract (BE)	Male Wistar rats, *n* = 24, 25 mg/kg/body weight/day, 17 weeks	Restored changes in gut dysbiosis induced by HF diet, ↑ *Rumminococcus*, *Pseudoflavonifractor*, Sporobacter and ↓ *Oscillobacter*	↓ tryptophan	↓ LPS, ↓ Tryptophan positively correlated to TCK-1 expression in the hippocampus, ↑	[78]
Blueberry anthocyanin-rich extracts (BAE)	Male C57BL/6 mice, *n* = 24, 100 mg/kg body weight/day or 200 mg/kg/body weight/day, 8 weeks	↑ *Bifidobacterium*, *Lactobacillus*, *Roseburia*, *Faecalibaculum*, *Parabacteroides* and *Ruminiclostridium*, and ↓ *Staphylococcus*	↑ Short chain fatty acid (SCFA) butyrate	↑ SOD and GSH-Px in the liver, ↑ 5-HT, ↑ dopamine, normalized neuron morphology	[79]
Polyphenol-rich blueberry-mulberry extract (BME), *Vaccinium uliginosum* L. and *Muros nigra* L.	Male C57BL/6J mice, *n* = 32, 300 mg/kg/d, 6 weeks	↑ *Lactobacillus*, *Streptococcus*, *Lactococcus*, *Corynebacteriaceae*, *Aerococcus*, *Enterococcus*, *Leuconostoc* and *Weisella*, ↓ *Blautia*, *Lachnoclostridium*, *Roseburia* and *Anaerotruncus*. Restored beta diversity	↑ 21 metabolites: fatty acids, amino acids, benzoid acids and indoles. *Blautia* positive correlation with methylcysteine, and negative with vanillic acid, *Lactobacillus* negative correlation with methylcysteine and positive with vanillic acid, *Streptoccocus* negatively correlated with methylcysteine and positively with EPA, linoleic acid and other fatty acids	Improved cognitive performance, ↓ neuronal loss, ↓ IL-6 and TNF-α levels in brain and intestine, ↑ levels of intestinal tight junction proteins (ZO-1 and occludin)	[86]
Anthocyanin rich extract *Rubus idaeus* (raspberries)	Male C57Bl/6J mice and APP/PS-1, *n* = 100, 100 mg/day (yellow or red raspberries), 24 weeks	↓ *Bacteroidetes*, ↑ *Proteobacteria*, No changes in bacterial richness	NC	No difference in cognitive functions, no improvement in microvascular architecture, modulated endogenous metabolites in brain/plasma	[118]
Grape-derived bioactive dietary polyphenol preparation (BDPP)	C57BL6/J male mice, *n* = 122, Grape seed extract 200 mg/kg/bw, resveratrol 400 mg/kg BW, grape juice polyphenol content 183 mg/kg BW, 13 days	↑ microbial α-diversity	NC	BDPP restored the SD-induced memory impairment. microbiota dysbiosis ↓ efficacy of dietary polyphenols, ↓ bioavailability of BDPP-derived phenolic acids	[119]
Flavanol-rich preparation (FRP) (cocoa)	Humanized gnotobiotic mice (FMT), *n* = 13, 40 mg FRP flavanol/kg BW/day, 15 days	Unique bacterial composition from human donors (HuA and HuB) with *Bacteroides ovatus*, *Bacteroides thetaiotanomicron*, *Bacteroides uniformis* and *Eggerthella lenta*	↑ DHCA and 3-HPPA plasma levels, unique phenolic acid metabolites in the caecum	↑ bioavailability of plasma-circulating DHCA and brain-accumulating 3-HPPA and 3,4-diHBA, linked to ↓ of Aβ misfolding, ↓ inflammation and ↑ in brain resilience	[120]
Citrus limon polyphenols (LPP)	Male SAMP1 mice, *n* = 36, 0.1% (*w*/*v*) LPP, throughout life	↓ Bacteroidetes/Firmicutes ratio	NC	↑ lifespan (3 weeks), ↓ ageing-related scores (e.g., peri-ophthalmic lesions) and locomotor atrophy	[121]
Coffee cherry husks (CCHP)	Female C57BL/6 J mice, *n* = 18, low dose 10 mg/kg, high dose, 30 mg/kg 0.5, 1, 7 days	↑ *Bacteroides* and *Bacteroidota*, ↓ *Allobaculum*, *Helicobacter*, and *Enterococcus*	NC	Restored inflamed gut microbiome, ↓ TNF-α, IL-1β, IL-6 and Cox-2, inhibition of TLR4/Myd88/NF-κB signalling pathway	[122]
Hawthorn fruit	Female KM female mice, *n* = 72, 100, 200, 400 mg/kg/d, 35 days	↑ *Dubosiella*, *Alloprevotella*, and *Bifidobacterium* ↓ *Acinetobacter*, *Akkermansia*, *Lachnospiraceae_NK4A136_group*, and *Staphylococcus*, ↑ richness and diversity	↑ Docosapentaenoic acid (DPA), sphingolipid (SM), phosphatidylcholine (PC), phosphatidylethanolamine (LPE) and lysophospholipid (LPC), ↓ succinic acid, hexadecanedioic acid, tetradecanedioic acid, 2-butoxyacetic acid, l-ascorbic acid 2-sulfate, and (R)-3-hydroxy myristic acid	↑ cognitive function, ↓ Aβ1–42 level in the hippocampus, inhibited abnormal activation of microglia, changed serum metabolites related to microbiota. ↓ MDA and ↑ enzymatic activity (SOD and GSH-Px)	[80]
*Dendrobium nobile* Lindl. (*D. nobile*)	Female Kunming mice, 200 mg/kg/bw, 8 weeks	Improved gut microbiota dysbiosis and reversed age-related changes in microbiome (reversed *Firmicutes*)	NC	↑ SOD, CAT and GSH-Px activities in the blood, and SOD and GSH-Px activities in the brain (↑ antioxidant activities), regulates endocrine system pathway genes, improved pathological tissue changes, positively affects gene expression levels related to ageing	[81]
Peanut shell (PS), *Arachis hypogaea* L. fruit	Male ICR mice, *n*= 120, low 100 mg/kg/day, medium 300 mg/kg/day and high dose 900 mg/kg/day, 2 weeks	↓ inflammatory response in the small intestine, ↑ alpha-diversity of gut microbiota, ↑ *Lachnospiraceae*	NC	↓ depression-like symptoms, ↓ inflammatory responses in the brain, in serum, and in small intestine, regulation of gut barrier tight-junction proteins, ↓ IL-1β, IL-6, TNF-α, in cortex and hippocampus	[123]
*Astragalus membranaceus*	C57BL/6J mice, *n* = 26, high dose 50 mg/kg/day, medium dose 25 mg/kg/day, low dose 5 mg/kg/day, 16 weeks	↑ species richness, ↑ butyrate-producing bacteria	NC	↓ fasting blood glucose, ↓ Aβ aggregation in the brain, ↑ expression of PSD95 and synapsin, positively modified mitochondrial biogenesis in the hippocampus, protected BBB and gut barrier. ↓ inflammatory cytokines and LPS	[124]
*Ficus pandurata* Hance var. angustifolia, Ficus of Moraceae	Male C57BL/6J mice, *n* = 30, 0.1% (*w*/*w*), 4 weeks	↓ *Firmicutes*. ↑ *Aerococcus*, *Bifidobacterium*, *Faecalibacterium*, *Bacteroides*, *Akkermansia*, *Allobaculum*, and *Prevotella*. ↓ *Pediococcus*	↑ actinonin, 4-methylumbelliferone, genioin, decosahexaenoic acid ethyl ester (DHA-ee) and enoxacin and ↓ neuropathic metabolites (stearoylcarnitine, 2-monolinolein, 4-hydroxybutyric acid and benzenoids). ↓ secondary bile acids, cholesterol metabolism and isoflavonoid biosynthesis. ↑catecholamine transferase inhibitor, caffeine metabolism and isoflavone biosynthesis	Improved exploration and memory behaviours. ↑ intestinal barrier functions, ↑ expression of occludin, ↓ expression of Aβ in the hippocampus, ↓ IL-6 level	[84]
Seabuckthorn (*Hippophae rhamnoides* L.)	Male adult ICR mice, *n* = 24, 20 mg/kg/day or 100 mg/kg/day, 14 days	Normalized Firmicutes levels, ↓ *Lactobacillaceae*, ↑ *Lachnospiraceae*, regulated gut microbiome. ↑ *α-diversity*	NC	Restored CUMS-induced damage to the hippocampus, ↑ levels of neurotransmitters, ↑ levels of neurotrophins, ↓ expression of IL-1β, IL-6 and TNF-α in cortex and hippocampus. Positive correlation between *Lachnospiraceae* and neurotransmitters and negative with inflammation and stress-hormones	[125]
*Acanthopanax senticosus* (AS)	Male KM mice, *n* = 35, 250 mg/kg/d, 1, 3,7, 14 and 28 days	Changed microbiota composition, ↓ Helicobacter, ↑ *Lactobacillus*, *Ruminococcaceae*, *Peptosreptococcaceae*, *Clostridiales_vadinBB60_group* and *Porphyromonodaceae*	NC	Prevented learning and memory loss, ↑ tight junction protein, ↑ expression of BDNF and NF-κB, maintained hippocampal neurons, restored GABA levels, ↑ 5-HT. Positive correlation between 5-HT and Lactobacillus and Ruminococcaceae	[126]
Nopal (*Opuntia ficus indica*)	Wistar rats, *n* = 25, 5% nopal water content, 7 months	↑ alpha diversity, ↑ *Ruminococcus bromii*, *Rumminococcus flavefaciens*, *Lactobacillus reuteri*, *Bacteroides fragilis* and *Akkermansia muciniphila*. ↓ *Bacteroides acidifaciens*, *Blautia producta*, *Faecalibacterium prausnitzii*, *Butyricicoccus pullicaecorum* and *Clostridium citroniae*	NC	Restored the mucus layer, improved cognitive functions, ↑ abundance of occludin, ↑ intestinal permeability, ↓ LPS serum levels, ↓ expression of proinflammatory genes Tnf-α, and NADPH oxidase, ↓ brain malondialdehyde (MDA) concentration, ↓expression of inflammation and oxidative stress in adipose tissues	[85]
Polyphenol blends/mixtures					
Xanthohumol, quercetin and phlorotannin extracts	Sprague Dawley rats, *n* = 54, X 10/mg/kg/day, Q 20 mg/kg/day and P 20/kg/day respectively, 8 weeks	Change in ß diversity, ↑ *Enterorhabdus*, *Asteroplasma*, *Lachnospiraceae* and *Coprococcus*	↑ BCFAs, isobutyrate and valerate	Antidepressant and anxiolytic effects, ↓ corticosterone (xanthohumol), ↑ BDNF, 5-HT	[127]
Grape Seed Polyphenolic Extract (GSPE) and resveratrol	Male C57BL/6J mice, *n* = 36 GSPE, 0.4 g and resveratrol 0.4 g in water, 2 weeks	↓ *Firmicutes*, ↑ *Bacteroides*, *↓ Clostridium*, ↑ *Parasutteralla* and *Akkermensia*	NC	↑ locomotor response, mitigated behavioural response to opioids	[128]
Chlorogenic acid (CGA) and epigallocatechin-3-gallate	Female ICR mice, *n* = 35, 20 mg kg^−1^ d^−1^ chlorogenic acid, 20 mg kg^−1^ d^−1^ EGCG, or 20 mg kg^−1^ d^−1^ chlorogenic acid plus 20 mg kg^−1^ d^−1^ EGCG, 8 weeks	↓ *Firmicutes/Bacteroides*, ↓ *Lactobacillaceae*, *Erysipelotrichaceae*, *Deferribacteraceae*, ↑ *Lachnospiraceae*, *Muribaculaceae*, and *Rikenellaceae*	NC	↓ gut permeability, ↓ endotoxemia and colon inflammation markers. Combination CGA plus EGCG recovered moving ability, ↓ gut inflammation, ↓ reactive oxygen species accumulation	[104]
Triphala polyherbal formulation (*Emblica officinalis*, *Terminalia chebula*, and *Terminalia bellerica mixture)*	APP/PS1 mice, *n* = 30, 250 mg/kg (extract) and 500 mg/kg (Powder)/day, 60 days	↓ gut dysbiosis, ↑ *Verrucomicrobia*, *Bacteroidetes*, *Proteobacteria*, *Actinobacteria*	↑ SCFAs acetic acid, propionic acid and butyric acid	↑ cognitive functions, ↓ LPS level, anti-inflammatory parameters (TNF-α, IFN-γ and IL)	[129]
Triphala polyherbal formulation (Emblica officinalis, Terminalia chebula, and Terminalia bellerica mixture)	5XFAD mice, *n* = 45, 500 mg/kg twice a day, 60 days	↓ *Cyanobacteria*, ↑ gut transition time	↑ SCFAs butyrate levels	↑ learning and memory function, mitigates dysbiosis in prolonged antibiotics treatment	[130]
Quercetin and 2-hydroxypropyl-B-cyclodextrin	C57BL/6 J mice, *n* = 18, 40 mg/kg/d of Quercetin complex, 6 days	↑ *Firmicutes*, ↓ *Bacteroidota*, reversed the changes in the relative abundance	NC	↑ spontaneous activity behaviour and short-term memory ability as well as anxiety level, ↓ TNF-α and IL-6 levels, and ↓ intestinal and hippocampal inflammation	[131]
Hizikia fusiforme, Brown algae—Polyphenol Polyssacharide Complex (PPC)	Kunming mice, *n* = 108, 10 mg/mL PPC, 37 days	Changed intestinal flora diversity, ↑ Firmicute/Bacteroidetes (F/B) ratio	NC	Activate the Nrf2-ARE signal pathway, and related antioxidant pathways (Nqo1 and SOD1) in brain mice, ↓ MDA and improved LPO clearance rate	[82]
Polyphenol blend (citrus pulp, carrot, and spinach) and fish oil	Dogs, *n* = 40, Blend of test food with 106 mg/g polyphenols and lycopene 0.054 ppm, fish oil (0.5%), 30 days	↑ *Coxiellaceae*, *Blautia*, *Parabacteroides*, *Eubacterium*, *biforme*, *Acholeplasma*, and *Odoribacter*. ↓ *Flexispira* and *Gammaproteobacteria*	↓ 4-EPS and sphingomyelin levels in serum, ↑ azelate and choline in faeces	4-Ethylphenyl sulphate negatively correlates with metabolites related to anxiety-like disorders	[132]
Symbiotic					
Grape-derived Bioactive dietary polyphenol preparation (BDPP) with *Lactobacillus plantarum* and *Bifidobacterium longum*	C57BL/6J male mice, *n* = 43, 1% *w*/*v* resveratrol, 1% *w*/*v* grape seed polyphenol extract, and 5% *w*/*v* concord grape extract	↑ SCFAs producing bacteria	↑ plasma and brain bioavailability of microbial-derived phenolic metabolites, ↑ polyphenolic and tryptophan metabolites	↓ chronic-stress inflammatory responses (ileum and prefrontal cortex), ↑ brain resilience, ↓ IL-6, TLR, IL-1 (symbiotic)	[133]
Nanoparticles					
Chlorogenic acid (CGA), nano system with selenium	APP/PS1 transgenic mice, *n* = 40, 80 mg/kg body weight/day, 16 weeks	↑ diversity and richness of gut microbiota. ↑ *Turicibacter*, *Colidextribacter*, *Ruminococcus*, *Alloprevotella*, and *Alistipes*. ↑ *Bacteroidetes*	NC	↓ Aβ aggregate-related neuroinflammation and glucose homeostasis disorder in the brain, ↑cognitive impairment, ↓ oxidative stress	[134]
Polyphenol-armoured chitosan and tannic acid (CHI/TA)	Female C57BL/6J mice, *n* = 102, 1 mg mL^−1^, 8 days	Retains relative abundance of *Lactobacillaceae*, *Muribaculaceae* and *Bifidobacteriaceae*. ↓ *Enterococcaceae*. Prebiotic activities modulated gut microbiota diversity and homeostasis	NC	↑ learning and cognitive abilities, ↓ anxiety-and depression-like behaviours and cognitive impairment. Inhibited expression of GABA receptors	[135]
Resveratrol (Res), selenium/chitosan	ICR mice, *n* = 40, 50 mg/kg body weight or 60 mg/kg body weight, 24 weeks	Regulation of *Entercoccus*, *Colidextribacter*, *Rikenella*, *Ruminococcus*, *Candidatus_Saccharimonas*, *Alloprevotella and Lachnospiraceae_UCG-006*. ↑ alpha-diversity. Regulated F/B ratio to normal levels, ↑ gut bacteria linked to ↓ antioxidation, lipid deposition and anti-inflammation	NC	Inhibited lipid deposition, ↓ oxidative stress and neuroinflammation, ameliorated glucose tolerance, ↓ MDA	[105]
Dietary pattern					
Pre-Hispanic Mexican diet (PMD), Diet rich in fibre, polyphenols, a healthy ratio of omega 6/omega 3 fatty acids, vegetable protein rich in β-carotenes, polyphenols, lycopene,	Male Sprague–Dawley rats, *n* = 24, Food combination (nopal, polyphenols, omega 3), 3 months	↓ *Fimicutes*, ↑ *Bacteroidetes*, *Bifidobacteria* and *Lactobacillus*	NC	↑ cognitive functions, ↓ glucose intolerance, serum and liver triglycerides and leptin, ↓ hepatic levels of ROS, oxidized proteins and GSSG/GSH ratio, ↓ MDA levels, ↓ adiponectin	[106]

Abbreviations: Non-communicated (NC), Short-chain fatty acids (SCFAs), Branched-chain fatty acids (BCFAs), Malondialdehyde (MDA), Amyloid Beta (Aβ), Lipid peroxidation products (LPO), Reactive oxygen (RO), Reactive oxygen species (ROS), *O*-Desmethylangolensin (*O*-DMA), Brain-derived neurotrophic factor (BDNF), Tumour necrosis factor (TNF), Interleukin (IL), Toll-like receptors (TLRs), Lipopolysaccharides (LPS), Firmicutes/Bacteroidetes (F/B), 5-hydroxytryptamine receptors (or serotonin receptors, 5-HT).

### 3.2. Clinical Evidence

With regard to the clinical evidence, there is a marked lack of data, with only three human clinical studies considering the mediating effects of (poly)phenols on the gut–brain axis to date, and a notable absence of molecular mechanisms (Table 2). In addition, the current available studies reflect an emphasis on (poly)phenol-rich foods rather than isolated compounds. As far as studies recognising the effects of (poly)phenols on microbiome structure, metabolites and related functions, it is challenging to draw conclusions due to the paucity of available studies as well as the heterogeneity in design and trial duration. The clinical study conducted by Romo-Vaquero [136] in Parkinson’s disease patients shows a correlation between a decrease in gut diversity favouring a pro-inflammatory bacterial composition and a decreased production of anti-inflammatory metabolites (urolithin) as well as the SCFA butyrate. This translates into increased production of *p*-cresol and lipopolysaccharides (LPS), as well as increased degradation of intestinal mucus, suggesting enhanced permeability of the gut cell wall. Of interest here, the uraemic toxin *p*-cresol sulfate (pCS) is known to increase endothelial permeability and has been recently linked to cerebrovascular damage due to its disruptive actions on BBB integrity [61,137]. Other studies, although lacking the molecular mechanisms linked to the GBA and related derived metabolites, have evaluated the effects of olive oil polyphenols, major components of the Mediterranean diet (Med Diet). Casamenti and Stefani highlighted the prebiotic effects of (poly)phenols present in extra virgin olive oil (EVOO) in a subset of the PREDIMED trial, and their cognitive benefits [138,139]. Of interest, the Med Diet is known to preserve cognitive functions and reduce the risks of neurological diseases with effects ranging from preserved brain volumes and cognitive abilities [140], reduced cognitive decline [141] and overall inflammation during ageing [142]. However, further research is needed to investigate the impacts of polyphenols present in EVOO on the GBA and their related metabolites.

**Table 2:** The effects of polyphenols on gut microbiome structure, microbial metabolites, and related brain functions in models of neurological disease and ageing: Results from Clinical studies.

Polyphenol Type	Study Model, Sample Size, Dose, and Duration	Changes in Microbiome Structure	Changes in Microbial Metabolites	Functions	Refs.
Ellagitannins					
Polyphenols from walnuts	Parkinson’s disease patients, *n* = 52 30 g of walnuts, 3 days	↑ *Enterobacteriaceae*, *Desulfovibrionaceae*, *Lactobacillaceae*, *Enterococcaceae*, *Actinomycetaceae*, and *Olsenella* in PD patients. ↓ Ruminococcaceae, *Lachnospiraceae*, *Faecalibacterium*, ↓ urolithin-producing bacteria	↓ Urolithin production in PD patients, ↓ anti-inflammatory metabolites, ↓SCFA butyrate, ↑ p.cresol production	↑ LPS, ↑ intestinal mucus breakdown, ↑ tyrosine degradation, correlation with health-related microbial biomarkers	[136]
Anthocyanins					
Wild blueberry (WB)	Dietary intervention, *n* = 61, 302 mg anthocyanins, 12 weeks	No change in gut microbiota composition	NC	↑ vascular and cognitive function, ↓ 24 h ambulatory systolic BP	[143]
Flavanones					
Flavonoid-Rich Orange Juice	Depressive adults, *n* = 40, Daily 380 mL, 600 ± 5.4 mg flavonoids, 8 weeks	↑ *Lachnospiraceae*, *Eubacterium*, *Roseburia*, *Coprococcus*, *Agathobacter*, *Bifidobacterium* and *Bacteroides*	NC	Serum BDNF level significantly positively correlated with abundance of the *Lachnospiraceae*, and *Gemella* with homocysteine levels and depression	[144]

Abbreviations: Non-communicated (NC), Lipopolysaccharides (LPS), Parkinson’s disease (PD), Blood pressure (BP), Brain-derived neurotrophic factor (BDNF).

## 4. Conclusions

A growing body of evidence from preclinical and, to a lesser extent, clinical studies, confirm the effects of various dietary (poly)phenols on brain health and ageing, acting through modulation of the GBA. However, several key points pose challenges in understanding the role of (poly)phenols and GBA. For instance, although (poly)phenols have independently demonstrated their potential in gut health and in neuronal protection, studies reporting the direct modulation of gut–brain axis and the molecular mechanisms underlying these effects are very limited. While promising, clinical studies are currently scarce, therefore limiting information regarding the translational potential of these dietary products. The need for studies and human trials that are targeted, homogeneous and with reproducible results is therefore important [60]. In this respect, future studies should focus on the mechanisms to capture the complex interaction of these food bioactives with the gut microbiota and with host systems. In addition, because ageing of the gut is generally accompanied by a decrease in microbial diversity, which is a key factor in maintaining overall gut health and functionality, forthcoming studies should investigate the older segment of the population. Emphasis should be placed on characterising the brain’s health and its related systemic biomarkers, as well as on neuroimaging, coupled with analysis of the gut microbiome, to better understand the protective role of these natural compounds on healthy brain ageing.
